# Embryotoxicity and Teratogenicity of Steroidal Saponin Isolated from *Ophiopholis mirabilis*

**DOI:** 10.3390/toxics11020137

**Published:** 2023-01-30

**Authors:** Qian Xu, Xiao Yang, Ranran Zhang, Yaxi Li, Zhi Yan, Xiaodong Li, Bing Ma, Yanfang Liu, Ainuo Lin, Shaoshuai Han, Ke Li, Li Chen

**Affiliations:** 1 Jiangsu Provincial Institute of Marine Resources Development, College of Pharmacy, Jiangsu Ocean University, Lianyungang 222005, China; 2 Yantai Institute of Coastal Zone Research, Chinese Academy of Sciences, Yantai 264003, China; 3 College of Resources and Environment, University of Chinese Academy of Sciences, Beijing 100049, China; 4 School of Ocean, Yantai University, Yantai 266071, China; 5 School of Marine Science and Technology, Harbin Institute of Technology, Weihai 264209, China

**Keywords:** *Ophiopholis mirabilis*, secondary metabolite, isolation, zebrafish, embryotoxicity

## Abstract

Benthic invertebrates produce secondary metabolites that serve as defenses against consumers and promote their fitness. To explore the chemical defense in marine benthic echinoderms, the chemical constituents of *Ophiopholis mirabilis* were investigated. A steroidal monoglycoside, asterosaponin P1, was isolated from *O. mirabilis* for the first-time using column chromatography. The chemical structure was characterized by spectroscopic and spectrometric methods. The embryotoxicity and teratogenicity of the isolated compound were assessed using the zebrafish embryo assay, a powerful vertebrate animal model system to study mechanisms of toxicity. When applied at high concentrations, asterosaponin P1 causes a significant increase in embryo mortality. A moderate LC_50_ of asterosaponin P1 appeared to be time- and concentration-dependent in its toxicity to zebrafish embryos. Teratogenicity in zebrafish embryos also included morphological defects, decreased hatchability, and a reduced heart rate. These findings revealed that steroidal saponin extracted from *O. mirabilis* exhibited acute toxic effects on zebrafish embryos, suggesting a potential chemical defense function in marine habitats.

## 1. Introduction

The brittle star *Ophiopholis mirabilis* belongs to Echinodermata, Ophiuroidea, Gnathophiurina, Ophiactidae, Ophiopholis, is an important contributor to biomass in benthic ecosystems [1]. The Ophiuroidea, which account for nearly one-third of all species of extant echinoderms, are the largest group [2]. All seas have dense aggregations of brittle stars, which have evolved a variety of lifestyles and can be found from the intertidal zone to the deepest levels [3,4]. The majority of species live in crevices and holes in coral or rock, submerged in muck, or at the bottom of the ocean [2]. *O. mirabilis* is a widespread species that aggregates at the bottom of the Yellow Sea and is found along the western coasts of the northern Pacific Ocean [5,6]. *O. mirabilis* is an epizoic organism that inhabits numerous hosts such as sponges, cnidarians, ascidians, echinoderms, algae, bryozoans, and seahorses without camouflage in the southwest Atlantic [7]. Moreover, gorgonians and sponges are *O. mirabilis*’ primary hosts in the eastern Pacific [8]. Brittle stars distinguish themselves sharply from many host species in terms of color and visibility. They might therefore be born with an innate predator deterrent, such as chemical defenses. Their secondary metabolites, which have acute toxicity in the aquatic ecosystem, might be the potential chemical deterrent to effectively enhance their survival.

Previous chemical investigations of animals of the class Ophiuroidea have isolated several polyhydroxysteroid sulfates and saponins as the main classes of secondary metabolites [9,10,11,12,13]. Many of the steroid sulfates and crude extracts exhibited a broad spectrum of biological activity, including cytotoxicity [14], hemolytic activity [15], and repellant activity to other marine organisms [16]. Although the parameters for the extraction of saponin [17] and pepsin-soluble collagen [18] were optimized in *O. mirabilis*, there is no report on the secondary metabolites. Recent research has shown how saponin affects the hemolysis of red blood cells and can even be hazardous to some animal species [19,20]. To investigate toxicity mechanisms, zebrafish (*Danio rerio*) can be used as a robust vertebrate animal model system, which is a popular alternative to mammalian animal testing [21]. Due to its efficacy in both experimental and genetic analyses, zebrafish, as a model of conventional vertebrate development and disease pathogenesis, has attracted increased attention from the scientific community [22,23].

In this study, we investigated the putative chemical defense in *O. mirabilis* collected from the Changdao archipelago, the interface between the Yellow Sea and the Bohai Sea of China. The secondary metabolite was characterized, and its toxic effects on early vertebrate embryonic development were assessed using a zebrafish embryo assay. During the first four days of zebrafish growth, the effects of various steroidal monoglycoside sulfate concentrations were investigated with regard to mortality and morphological alterations. The research highlights the potential broad-spectrum chemical defenses of echinoderms by employing the zebrafish as a model animal and emphasizing the ecological role of secondary metabolites in marine ecosystems.

## 2. Materials and Methods

### 2.1. Sampling

The *O. mirabilis* (20.5 kg) was collected in Changdao Sea Area, Shandong, China (37°55′12″ N, 120°43′48″ E), in June 2020. The sample with the vibrant hue was chosen for further chemical investigation. A voucher specimen was deposited in our laboratory at −20 °C with reference number C2020-002.

### 2.2. Extraction and Isolation of Secondary Metabolites

The air-dried and ground *O. mirabilis* (20.5 kg) was extracted for 3 to 72 h at room temperature with 95% ethanol/water. After the solvent was removed under reduced pressure at <40 °C, a dark residue was obtained. The residue substance was suspended in water and then subsequently partitioned using petroleum ether, ethyl acetate, and n-butanol. According to the TLC analysis result, the n-butanol extract (172.3 g) was chromatographed over silica gel (1500 g), eluting with CH2Cl2–MeOH to give 15 fractions. Column chromatography (CC) was performed on silica gel (100–400 mesh, Qingdao Marine Chemical Factory). Fr. 10 (20.3 g) was further fractionated by common chromatography on silica gel eluting with a gradient of increasing MeOH (20–100%) in CH2Cl2 to yield two subfractions. A compound (4.5 mg) was produced by chromatographing the second sub-fraction twice over Sephadex LH-20 and eluting it with MeOH-H2O (1:1) and MeOH, respectively.

### 2.3. Chemical Structure Elucidation Procedures

On the AVANCE 500 MHz instrument (Bruker, Billerica, MA, USA), ^1^H and ^13^C/DEPT-NMR, two-dimensional homonuclear (i.e., COSY), and heteronuclear (i.e., HMQC and HMBC) analyses were carried out. Mass spectrometry was performed on a Waters ACQUITY H-Class UPLC system tandem a Waters Xevo TQ-S triple quadrupole time-of-flight mass spectrometer (Waters Corp., Milford, MA, USA).

### 2.4. Zebrafish Embryo Bioassay

The Yantai Institute of Coastal Zone Research, Chinese Academy of Sciences (2021R001) Animal Care Ethics Committee’s regulations were strictly followed when using animals in this study. Adult zebrafish breeding and embryo toxicity testing were carried out as instructed by Berry et al. [24,25]. Briefly, the zebrafish kept at 12/12 h light/dark conditions were placed in stainless mesh spawning cages in the morning with lights on (9:00–9:30 a.m.). Eggs that had been fertilized (4 h after fertilization, 4 hpf) were retrieved and then washed with egg water, a solution of sea salt with a conductivity of 500–550 s/cm. The preliminary experimental findings led to the maximal concentration of the chemical being established at 25.0 mg/L [26]. To examine the effects of the chemical on zebrafish at sub-lethal concentrations, 5.0, 7.5, 10.0, and 15.0 mg/L were tested. Using 12-well polypropylene plates, each treatment contained 4 wells with 12 embryos (48 embryos per treatment in 5 mL). Vehicle (DMSO-only) and “untreated” controls (egg water-only) were used in each assay; the solvent was removed from the assay plates before the medium and embryos were added, so no effects of the solvent were seen (compared to the untreated control). Up to 4 h post-ovulation, the embryos’ bioactivity was monitored. In addition to the percentage of hatching and mortality, the heartbeat of zebrafish embryos was recorded using photomicrography with an Olympus CKX53 digital camera for 20 s.

#### 2.4.1. Dose-Response to Zebrafish Embryos

Zebrafish embryo mortality is used to gauge a species’ embryotoxic potential. Successfully fertilized embryos are chosen under a microscope for embryo exposure trials 3–4 h after zebrafish spawn. According to the research group’s preliminary experiments, the concentrations of asterosaponin P1 were 5, 7.5, 10, 15, and 25 mg/L, respectively. Ten fertilized eggs from each concentration were treated and transferred to 12-well plates. A 96-hour exposure experiment was then conducted, and 10 embryos per well were exposed to 5 mL of solution. Every 24 h, embryos are examined under a stereomicroscope to check for signs of embryonic death, which include coagulation and/or no heartbeat. Each experiment is conducted four times. Mortality is determined based on the number of dead embryos at each concentration of 96 hpf.

#### 2.4.2. Zebrafish Embryo and Larvae Teratogenicity

After exposure to five different doses of asterosaponin P1, the teratogenicity of zebrafish was assessed by morphological changes and developmental abnormalities in both the embryos and the larvae. Every 24 h following treatment, the embryos and larvae were observed under a stereomicroscope. Pericardial edema, delayed yolk sac absorption, tail curvature, etc. are all regarded as abnormalities of embryonic development.

#### 2.4.3. Zebrafish LC_50_ and Mortality Calculations

Take 96 hpf as an example of statistical methods of mortality over time: 96 hpf mortality rate (%) = (total number of accumulated dead embryos in 96 hpf/total number of embryos in the initial test) × 100%, and so on. The original data for each test level was then used to generate preliminary statistics. The exposure dose of half embryonal lethal (LC_50_) lysins at various exposure time periods was determined using Probit analysis and SPSS 10.0 statistical software.

#### 2.4.4. Zebrafish Hatching Rate

Zebrafish embryos typically incubate for 48 to 96 hpf. According to the following statistical procedure, each period’s hatching rate is calculated. For instance, 96 hpf hatching rate (%) = (96 hpf hatching embryos total embryos) × 100%.

#### 2.4.5. Zebrafish Deformity Rate

Every time period’s aberrant phenotypes were recorded, and the corresponding ratio was calculated. Pericardial edema, yolk sac edema, spinal curvature, and body length decrease are phenotypic abnormalities that may manifest in embryos.

### 2.5. Statistics

Four independent replications of each treatment were used in the investigations on embryotoxicity and teratogenicity. One-way analysis of variance (ANOVA) and Tukey’s multiple comparison test were used to compare the mortality of zebrafish embryos and teratogenicity test results. The mean ± standard deviation of the mean (SD) was used to present all data, and *p* < 0.05 was considered significant.

## 3. Results and Discussion

### 3.1. Chemical Structure Elucidation of Asterosaponin P1

**Figure 1:**
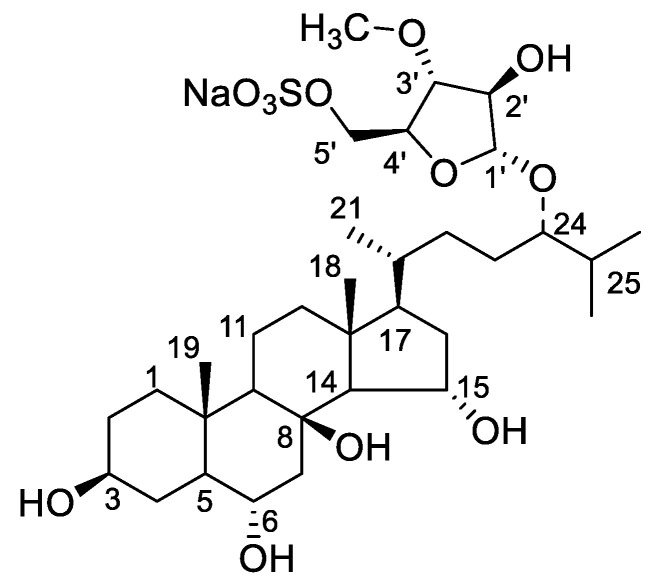
Chemical structure of asterosaponin P1.

The isolated compound (Figure 1) was obtained as a pale amorphous powder. The high-resolution negative ion mass spectral data gave a pseudomolecular ion of *m/z* 677.3580 [M − Na]^–^, corresponding to the molecular formula of C_33_H_57_NaO_12_S, (cal. 677.3571 [M − Na]^–^, Δ = 1.3 ppm).

**Figure 2:**
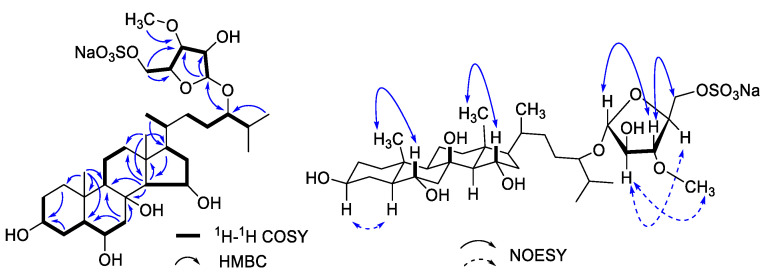
Key correlation of COSY, HMBC, and NOESY of asterosaponin P1.

1D- and 2D-NMR analysis was used to determine the compound. (Figures S2 and S3 and Table 1). Two methyl singlets representing the methyl groups C-18 and C-19 and three doublets for the methyl groups C-21, C-26, and C27 were detected in the ^1^H, ^13^C, and HSQC NMR spectra (Figures S2–S4). These methyl group indications pointed to a typical 27-carbon steroid skeleton, which was supported by the predicted chemical formula. Long-range communication between the methyl protons for Me-19 to a CH-5 (*δ*_C_ 53.7) was one of the crucial HMBC correlations. The COSY spectrum (Figure S5) showed correlations between CH-CH_2_-CH-CH-CH_2_ (Figure 2). The proton on C-14 also showed a COSY correlation to the CH at *δ*_H_ 4.21 (Figure 2), implying the oxygenation on C-15. The long-range communication between the methyl protons for Me-26 and Me-27 to carbinol methine proton at *δ*_C_ 84.7 allowed us to locate another site of oxygenation on C-24. These relationships were highly reminiscent of the hydroxylation pattern in asterosaponin P1 [27].

Accounting for the molecular formula deduced by HR-MS, we assume the composition of the C24 substituent to be a C_6_H_10_NaO_8_S subunit. A CH_2_ resonance at *δ*_H_ 4.12 was attached to a carbon having a chemical shift of *δ*_C_ 69.1 (HSQC). This is a characteristic set of chemical shifts for sulfated primary alcohol [28]. The COSY correlations of CH-CH-CH-CH-CH_2_ (Figure 2) constructed the monoglycoside moiety. In addition, the long-range correlations from H-1′ to C-2′ and C-3′, from H-5′ to C-3′ and C-4′, and from protons of oxygenated methyl to C-3′ confirmed the chemical structure of sugar moiety. Of note, the correlations between H-1′ to C-24 and H-24 to C-1′ in the HMBC spectrum confirmed the linkage of monosaccharide on C-24. Therefore, the planar structure was assigned as 5′-*O*-sulfate 24-(3-methyl-arabinofuranosyl)-3,6,8,15,24-pentaoxy-cholestane, consistent with asterosaponin P1 [27].

The NOESY (Figure 2) and coupling constant analysis supported the relative configuration in this case. The hydroxyls at C-3, C-6, and C-15 exhibited an equatorial orientation, according to the NOESY correlations detected from H-19 to H-6*β*, from H-15*β* to H-18, as well as on the counterpart, the correlations from H-3*α* to H-5*α*. Furthermore, in the NOESY spectrum (Figure 2), the correlations from H-1′ to H-3′, and from H-3′ to H-5′; on the counterpart, the H-2′ displayed a correlation with H-4′; the H-2′ also showed correlation with protons of oxygenated methyl, indicating the relative configurations on sugar subunit. Although the stereochemistry at C-24 is tentatively assigned as 24*S* by analogy with nodososide [29], the comparison of the chemical shifts presented an unambiguous answer to this question unless each of the C24-epimers has been synthesized. The chemical shifts of **1** show equal to the analogy of asterosaponin P1 and different from the desulfate analogy. Therefore, we concluded that the isolated compound is 5′-*O*-sulfate 24-(3-methyl-L-arabinofuranosyl)-3*ꞵ*,6*α*,8,15*α*,24-pentaoxy-cholestane, asterosaponin P1.

### 3.2. Zebrafish Mortality and Hatching Rates Treated by Asterosaponin P1

**Figure 3:**
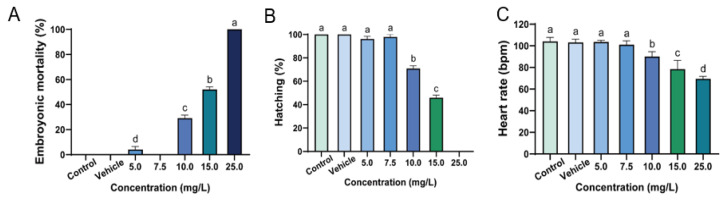
Cumulative mortality (**A**) and hatching efficiency (**B**) of zebrafish embryos exposed to asterosaponin P1 at 96 hpf, and heart rate (bpm) at 48 hpf (**C**), with a series of dilution concentrations of 5.0, 7.5, 10.0, 15.0, 25.0 mg/L, and 0.1% DMSO (vehicle). Zebrafish embryos’ coagulation and absence of a heartbeat were signs of mortality. Based on embryo deaths following exposure, percentages of embryonic mortality were estimated. Data represent the mean ± SD of four independent experiments (n = 12 embryos/group). Tukey’s multiple comparison test and one-way ANOVA were used to examine the results of the experiments. Significant differences across groups are denoted by different lowercase letters (*p* < 0.05).

Embryotoxicity was assessed at five different concentrations of asterosaponin P1 (5.0 mg/L, 7.5 mg/L, 10.0 mg/L, 20.0 mg/L, and 25.0 mg/L). The outcomes demonstrated that asterosaponin P1’s toxicity manifested in a concentration-dependent manner (Table S1). Figure 3A displayed the mean mortality of zebrafish at 96 hpf. The 0.1 DMSO (vehicle) caused no mortality in the zebrafish embryos. The lowest dose of asterosaponin P1 showed a significantly increased mortality rate (*p* < 0.05), although no viable mortality was observed in the groups treated with 7.5 mg/L of asterosaponin P1. The mortality rate of zebrafish embryos in the group treated with 10.0 mg/L, 15.0 mg/L, and 25.0 mg/L showed a significant increase. The sublethal endpoints (LC_50_) were 2.22 mg/L at 12 hpf, 23.33 mg/L at 24 hpf, 17.91 mg/L at 48 hpf, 14.58 mg/L at 72 hpf, and 13.35 mg/L at 96 hpf, respectively. Notably, higher doses of asterosaponin P1 were found to increase in embryotoxicity over the course of treatment (Table 2), suggesting that the toxicity of asterosaponin P1 appears to be concentration- and time-dependent manner in zebrafish embryos.

The hatching rate of zebrafish embryos treated with five different concentrations of asterosaponin P1 and 0.1% DMSO (vehicle) were presented in Figure 3B. All of the zebrafish embryos treated with control and vehicle were hatched successfully. As the treated concentration increased to 10.0 mg/L, the hatching rate of embryos showed a significantly decreased tendency. No hatching of any zebrafish embryos was observed in the 25.0 mg/L treated group.

The zebrafish heart rates at 48 hpf with different concentration treated were shown in Figure 3C. No significant difference between the means of the heart rates of the asterosaponin P1 (5.0 mg/L and 7.5 mg/L)-treated group and the 0.1% DMSO-treated group (vehicle). The average heart rate declined at the concentrations of 10.0 mg/L and above. The heartbeat decreased to 69.5 beats per minute (bpm) at 25.0 mg/L treated group, much lower than the normal heart rate of zebrafish embryos ranges from 120 to 180 bpm [30].

### 3.3. Teratogenicity of Zebrafish Embryos Treated by Asterosaponin P1

Saponins have some teratogenic effects on the growth and development of zebrafish embryos and juveniles [20]. No embryonic malformation was observed in the low concentration of asterosaponin P1 (≤5 mg/L). Zebrafish embryos were measured at various concentrations of asterosaponin P1 (5.0, 7.5, 10.0, 15.0, and 25.0 mg/L) across a range of exposure times (12–96 h) to analyze the saponin from *O. mirabilis* that caused teratogenicity. Table 2 displays the teratogenic consequences of asterosaponin P1 in zebrafish embryos. According to the findings, embryos exposed to astersaponin P1 at concentrations of 15.0 and 25.0 mg/L for 24 h had malformation rates of 10.76 ± 7.24% and 24.17 ± 9.36%, respectively. The results indicated that asterosaponin P1 was teratogenic to embryos at an early stage. Notably, at a concentration of 25.0 mg/L for 72 h, the greatest malformation rates were 90.25 ± 9.32%. The prolonged exposure period (96 h) at 25.0 mg/L resulted in no surviving embryo.

**Figure 4:**
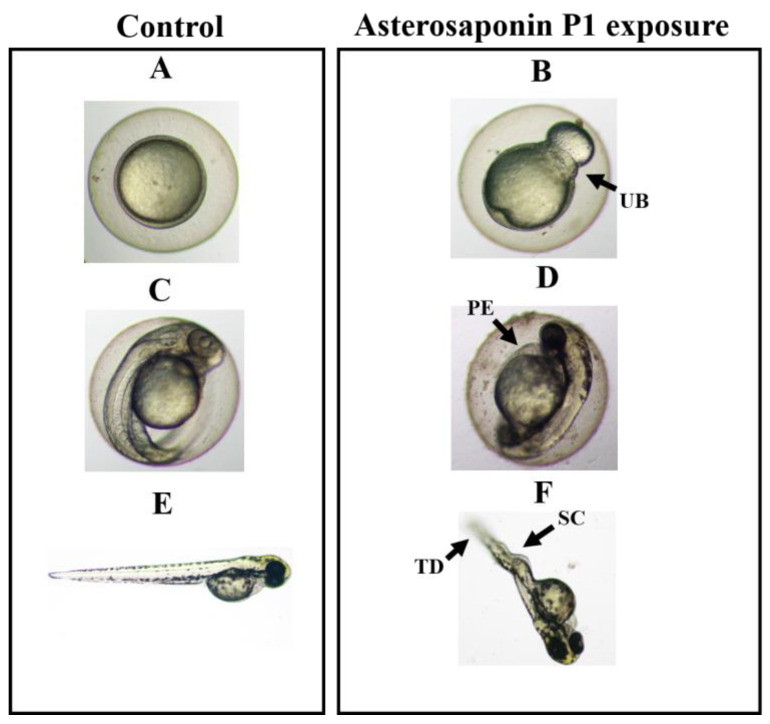
Effect of asterosaponin P1 from *O. mirabilis* on typical malformations in zebrafish embryos. The zebrafish embryos were exposed to 0.1% DMSO (vehicle), and 5.0, 7.5, 10.0, 15.0 and 25.0 mg/L of asterosaponin P1. The normal morphology of zebrafish embryo and larvae exposure to filtered water and to 0.1% DMSO (vehicle) at 12 h (**A**), 48 h (**C**) and 72 h (**E**). Typical malformations caused by 15.0 mg/L of asterosaponin P1 on zebrafish embryonic development at 12 h (**B**), 48 h (**D**) and 72 h (**F**). Description: embryo with teratogenic effect (**B**,**D**,**F**). Abbreviations: TD, tail extension deformity; SC, spinal column curving; PE, pericardial edema, and UB, unclosed blastopore. (Scale bars = 2 μm).

The morphology of the embryo was characterized by the presence of the unclosed blastopore (UB, Figure 4B), pericardial edema (PE, Figure 4D), and spinal curvature (SC, Figure 4F). Zebrafish embryos exposed to 25.0 mg/L at 12 h displayed an open blastopore (Figure 4B). The spinal abnormalities in the remaining embryos exposed to 15.0 mg/L ranged from 24.67 to 2.23% at 72 h and included caudal developmental problems (Figure 4F). A 100% mortality rate was observed after 96 h of exposure to 15.0 mg/L astersaponin P1. It’s interesting to note that neither the control group nor the 7.5 mg/L group of zebrafish embryos displayed any teratogenic abnormalities during the study (Figure 4A,C,E).

## 4. Conclusions

In our investigation of the chemical defenses in marine benthic echinoderms, asterosaponin P1 was isolated from brittle star *O. mirabilis* for the first time. Although brittle star merely contained a minute amount of asterosaponin P1, experiments on zebrafish embryos showed that it was teratogenic and embryotoxic, suggesting that it may have chemical defenses against nearby swimming predators. The toxicity of crude plant extracts containing saponins has been evaluated using the zebrafish embryos model [31,32,33], our study firstly provided the evidence that steroidal saponin isolated from marine invertebrates has a high impact on the growth and development of zebrafish embryos and had teratogenic effects on the morphology of zebrafish. Our findings highlight the chemoecological functions of steroidal saponins of echinoderms origin, providing insight into the chemical defenses of marine benthic echinoderms.

## Figures and Tables

**Table 1:** ^1^H, ^13^C NMR data of asterosaponin P1 (MeOH, *δ*/ppm).

No.	*δ*_H_ (*J* in Hz)	*δ*_C_	No.	*δ*_H_ (*J* in Hz)	*δ*_C_
1a	1.73 (m)	39.7 (CH_2_)	16a	1.90 (m)	42.0 (CH_2_)
1b	0.98 (m)		16b	1.78 (m)	
2a	1.73 (m)	31.6 (CH_2_)	17	1.35 (m)	56.2 (CH)
2b	1.47 (m)		18	0.97 (s)	15.6 (CH_3_)
3	3.48 (m)	72.3 (CH)	19	1.02 (s)	14.4 (CH_3_)
4a	2.19 (m)	32.5 (CH_2_)	20	1.33 (m)	36.5 (CH)
4b	1.20 (m)		21	0.90 (d, 6.5)	19.1 (CH_3_)
5	1.03 (m)	53.7 (CH)	22a	1.64 (m)	33.3 (CH_2_)
6	3.61 (m)	67.8 (CH)	22b	0.92 (m)	
7a	2.39 (dd, 13.5, 4.0)	50.4 (CH_2_)	23a	1.56 (m)	28.9 (CH_2_)
7b	1.37 (m)		23b	1.23 (m)	
8	--	76.2 (qC)	24	3.29 (m)	84.7 (CH)
9	0.87 (m)	57.4 (CH)	25	1.84 (m)	32.0 (CH)
10	--	38.0 (qC)	26	0.90 (d, 6.5)	18.3 (CH_3_)
11a	1.69 (m)	19.8 (CH_2_)	27	0.90 (d, 6.5)	18.7 (CH_3_)
11b	1.50 (m)		1′	4.94 (s)	109.7 (CH)
12a	1.95 (m)	43.0 (CH_2_)	2′	4.04 (m)	81.8 (CH)
12b	1.25 (m)		3′	3.59 (dd, 5.7, 2.4)	89.5 (CH)
13	--	45.6 (qC)	4′	4.19 (m)	82.0 (CH)
14	1.29 (m)	67.1 (CH)	5′	4.12 (d, 5.1)	69.1 (CH_2_)
15	4.21 (m)	69.9 (CH)	OMe	3.44 (s)	58.6 (CH_3_)

**Table 2:** Teratogenic effects of asterosaponin P1 from *O. mirabilis* on early development of zebrafish. The zebrafish embryos were treated to 0.1% DMSO (control), and 5.0, 7.5, 10.0, 15.0 and 25.0 mg/L of the asterosaponin P1 at different times of exposure (12, 24, 48, 72 and 96 h). Descriptive data represent the mean ± SD of teratogenic embryo percentage of four independent experiments (n = 40 per group). NE = no surviving embryo.

Concentrations (mg/L)	Times of Exposure (h)				
	12	24	48	72	96
0	0	0	0	0	0
5.0	0	0	0	0	0
7.5	0	0	0	0	0
10.0	0	0	24.67 ± 2.23	58.1 ± 8.57	82.30 ± 8.10
15.0	0	10.76 ± 7.24	39.26 ± 7.15	75.36 ± 3.94	NE
25.0	0	24.17 ± 9.36	49.23 ± 9.75	90.25 ± 9.32	NE

## Data Availability

Data are contained within the article and Supplementary Materials.

## Supplementary Materials

The following supporting information can be downloaded at: https://www.mdpi.com/article/10.3390/toxics11020137/s1, Figure S1: HR-ESI-MS of asterosaponin P1; Figure S2: ^1^H NMR spectrum of asterosaponin P1; Figure S3: ^13^C NMR and DEPT spectra of asterosaponin P1; Figure S4: ^1^H-^1^H COSY spectrum of asterosaponin P1; Figure S5: HSQC spectrum of asterosaponin P1; Figure S6: HMBC spectrum of asterosaponin P1; Figure S7: NOESY spectrum of asterosaponin P1; Table S1: Results of acute toxicity experiments of asterosaponin P1 on zebrafish embryos.
