# Supplementary material for: Embryotoxicity and Teratogenicity of Steroidal Saponin Isolated from Ophiopholis mirabilis
Source: Toxics. 2023 Jan 30;11(2):137. doi: 10.3390/toxics11020137 (PMC9959855; doi:10.3390/toxics11020137)
Supplement: Supplementary file 1 [file toxics-11-00137-s001.zip › toxics-2181117-supplementary.pdf]

toxics

Supplementary Materials: Embryotoxicity and Teratogenicity of Steroidal Saponin Isolated from *Ophiopholis mirabilis*

Qian Xu, Xiao Yang, Ranran Zhang, Yaxi Li, Zhi Yan, Xiaodong Li, Bing Ma, Yanfang Liu, Ainuo Lin, Shaoshuai Han, Ke Li and Li Chen

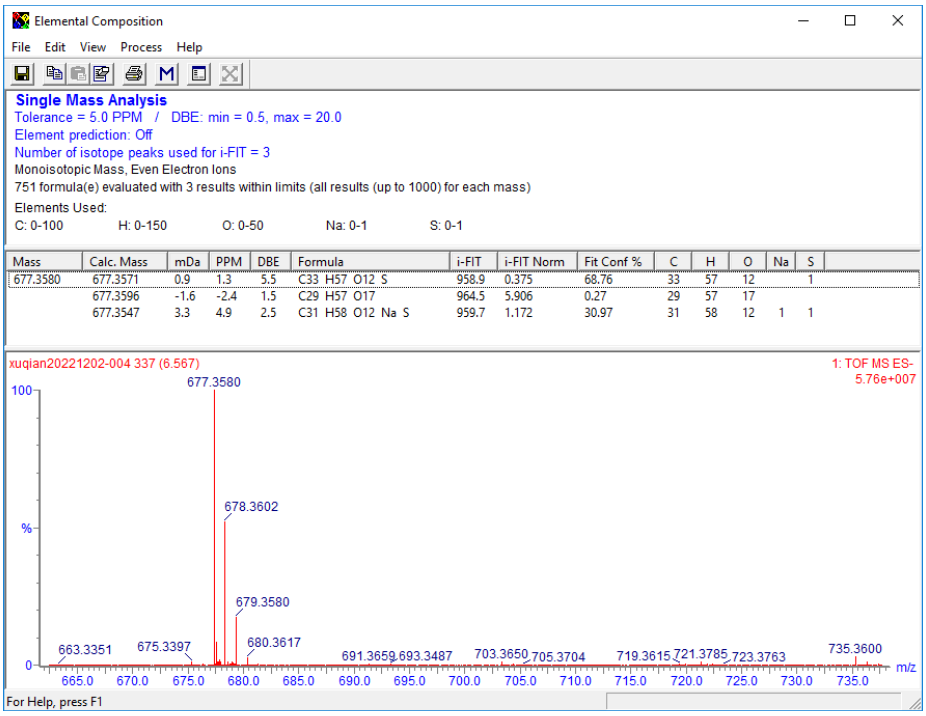

Figure S1. HR-ESI-MS of asterosaponin P1.

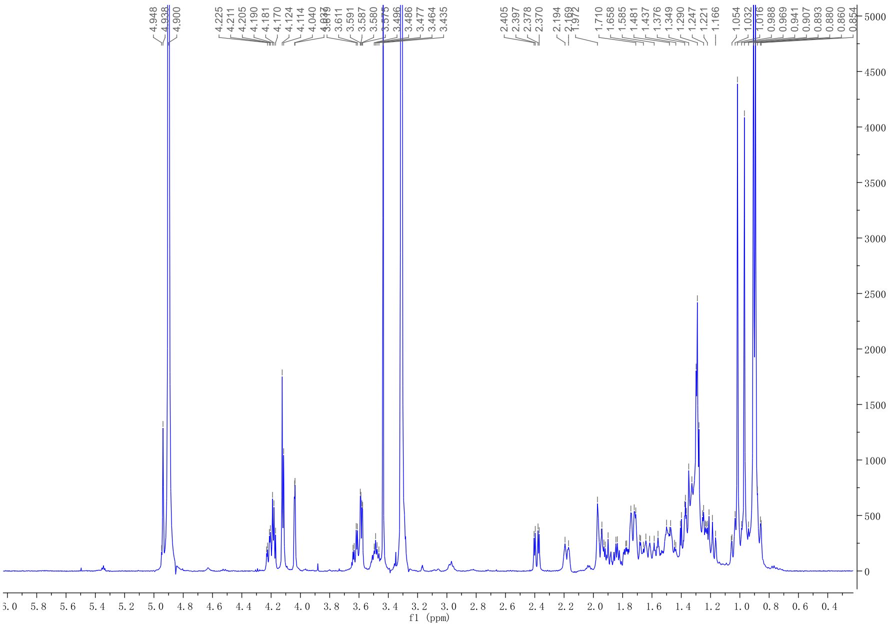

Figure S2. ^{1}H NMR spectrum of asterosaponin P1.

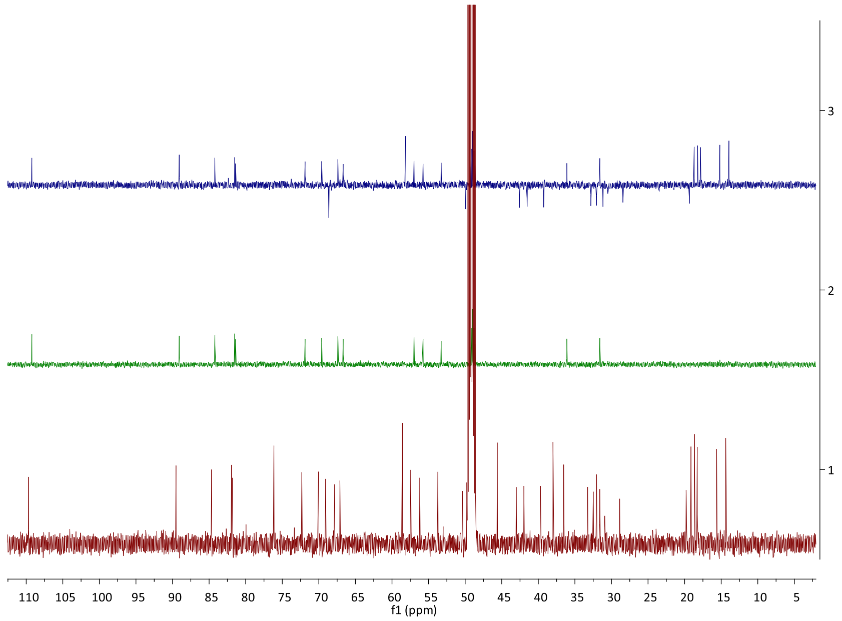

Figure S3. ^{13}C NMR and DEPT spectra of asterosaponin P1.

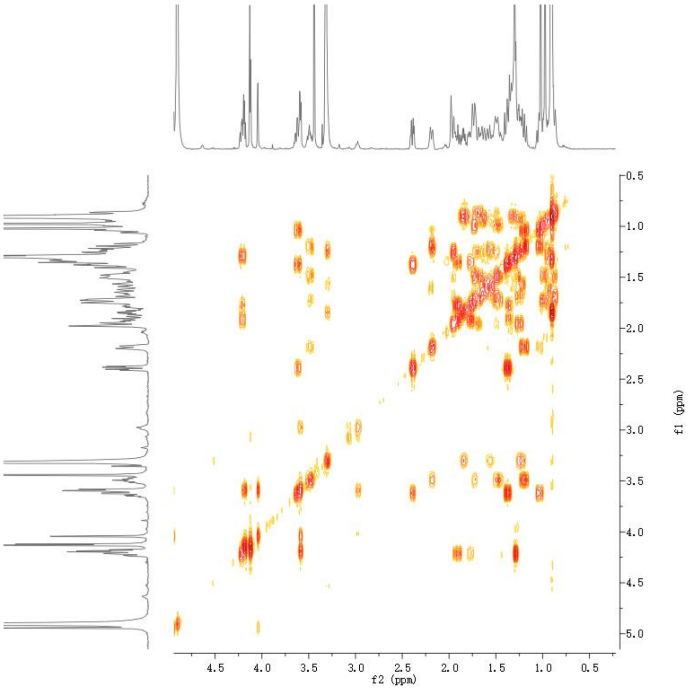

Figure S4. ^{1}H-^{1}H COSY spectrum of asterosaponin P1.

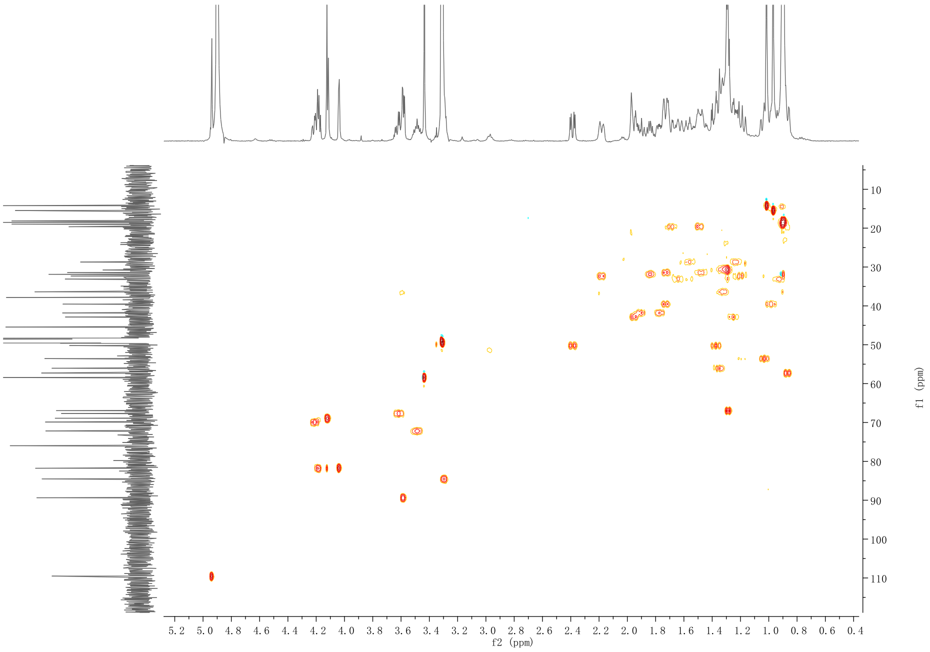

Figure S5. HSQC spectrum of asterosaponin P1.

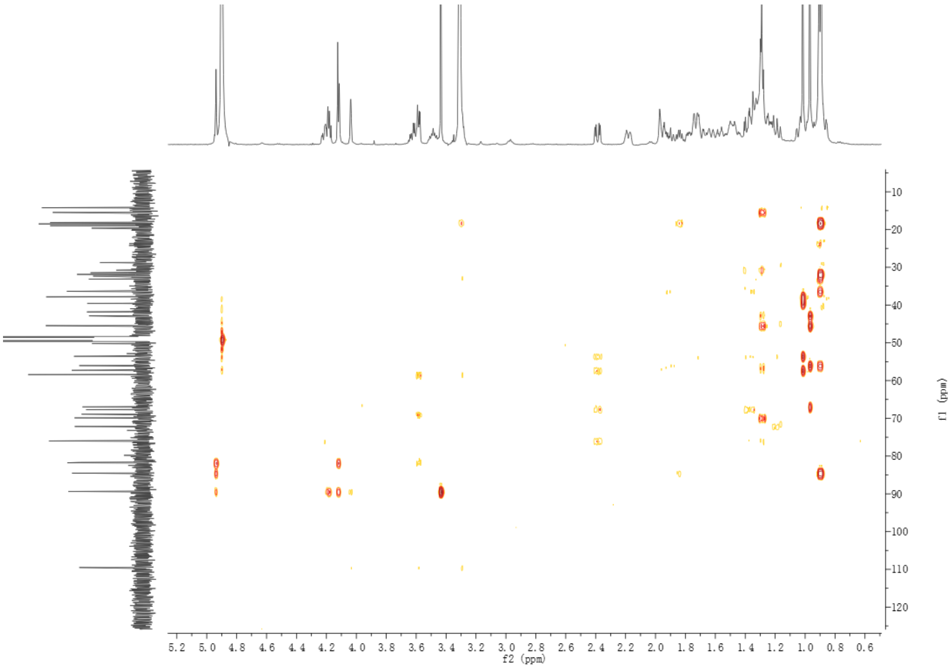

Figure S6. HMBC spectrum of asterosaponin P1.

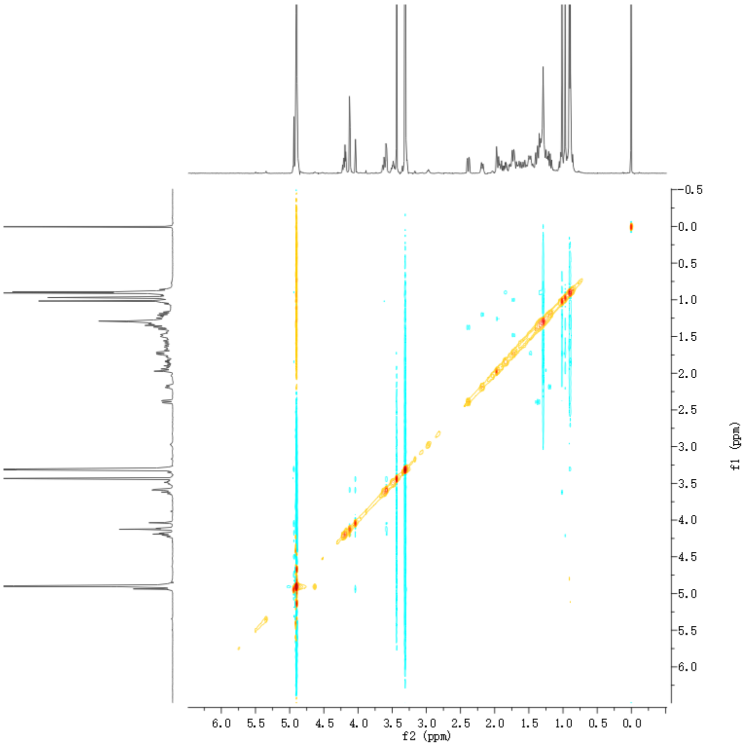

Figure S7. NOESY spectrum of asterosaponin P1.

Table S1. Results of acute toxicity experiments of Asterosaponin P1 on zebrafish embryos.

Time (hpf)	Mortality (%)							LC_{50} (mg/L)	R^2
	**0**	Vehicle	5.0 mg/L	7.5 mg/L	10.0 mg/L	15.0 mg/L	25.0 mg/L		
**12**	**0**	0	0	0	0	2.08	7.5	2.22	0.8213
24	0	0	3.21	9.35	14.58	38.22	45.53	23.33	0.9110
48	0	0	3.76	13.21	28.34	47.66	66.38	17.91	0.9526
72	0	0	3.92	15.62	32.93	49.95	89.25	14.58	0.9733
96	0	0	4.16	17.58	35.25	58.08	100	13.35	0.9733
